# Economic Analysis of the Reduction of Blood Transfusions during Surgical Procedures While Continuous Hemoglobin Monitoring Is Used

**DOI:** 10.3390/s18051367

**Published:** 2018-04-27

**Authors:** Borja Ribed-Sánchez, Cristina González-Gaya, Sara Varea-Díaz, Carlos Corbacho-Fabregat, Jaime Pérez-Oteyza, Cristóbal Belda-Iniesta

**Affiliations:** 1 Department of Manufacturing Engineering, ETSII-Universidad Nacional de Educación a Distancia (UNED), C/Juan del Rosal 12, 28040 Madrid, Spain; cggaya@ind.uned.es; 2 Department of Corporate Resources, HM Hospitales, Plaza Conde Valle de Suchil 2, 28015 Madrid Spain; 3 Department of Hematology, HM Hospitales, Plaza Conde Valle de Suchil 2, 28015 Madrid, Spain; svarea@hmhospitales.com; 4 Department of Anesthesia, HM Hospitales, Plaza Conde Valle de Suchil 2, 28015 Madrid, Spain; carloscorbacho@me.com; 5 School of Medicine, CEU San Pablo University, Urb. Montepríncipe, Ctra. Boadilla del Monte, Km. 5.300, 28925 Madrid, Spain; jperezoteyza@hmhospitales.com; 6 Department of R&D, HM Hospitales, Plaza Conde Valle de Suchil 2, 28015 Madrid, Spain; cbelda@hmhospitales.com

**Keywords:** spectrophotometry, photoplethysmography, real-time monitoring, transfusions, hemoglobin, cost savings

## Abstract

Background: Two million transfusions are performed in Spain every year. These come at a high economic price for the health system, increasing the morbidity and mortality rates. The way of obtaining the hemoglobin concentration value is via invasive and intermittent methods, the results of which take time to obtain. The drawbacks of this method mean that some transfusions are unnecessary. New continuous noninvasive hemoglobin measurement technology can save unnecessary transfusions. Methods: A prospective study was carried out with a historical control of two homogeneous groups. The control group used the traditional hemoglobin measurement methodology. The experimental group used the new continuous hemoglobin measurement technology. The difference was analyzed by comparing the transfused units of the groups. The economic savings was calculated by multiplying the cost of a transfusion by the difference in units, taking into account measurement costs. Results: The percentage of patients needing a transfusion decreased by 7.4%, and the number of transfused units per patient by 12.56%. Economic savings per patient were €20.59. At the national level, savings were estimated to be 13,500 transfusions (€1.736 million). Conclusions: Constant monitoring of the hemoglobin level significantly reduces the need for blood transfusions. By using this new measurement technology, health care facilities can significantly reduce costs and improve care quality.

## 1. Introduction

Blood transfusions are common practice in healthcare facilities, where up to 20% of patients who have undergone surgery and 35% of patients in intensive care units receive at least one blood transfusion [1,2].

Although necessary in terms of care and its quality, these transfusions involve significant costs. In 2008, four different studies were carried out in the United States which concluded that the costs of blood bags alone varied between $332 and $717 [3,4,5,6]. However, these studies only analyzed the cost of the blood bag, not the entire transfusion. For this, additional costs must be taken into account relating to the medical act of transfusion as well as conservation of bags, logistics, transport to and from centers, energy costs, compatibility tests prior to transfusion, and associated expendable material.

A few months after these studies were published, another study was carried out [7] based on activity-based costing (the ABC model) which analyzed and calculated all of the costs associated with transfusions in greater depth. The idea was to establish the total cost of a blood transfusion. Applying this new methodology, the costs related to transfusions increased considerably compared to the first studies and were more in the range of $522 to $1183, depending on the country studied. Also in 2008, Joseph Darbá published a study focused on Spain which concluded that the cost of a bag of blood was €150 and the cost of an entire transfusion was €350 [8]. 

Different criteria exist for determining the need to perform a blood transfusion, among which hemoglobin concentration in the blood plays a fundamental role. To obtain accurate levels of hemoglobin, analytical methods are traditionally used that are based on blood samples, which are analyzed in a laboratory at intermittent intervals. On occasion, the results from the analyses can take some time and do not show the evolution of the patient between the moment of extraction and when the analytical results are received [9]. In a stressful environment such as that of an operating theatre, these delays and uncertainties mean that some blood transfusions are unnecessary, especially in cases of stable anemia or perceptible but not very significant blood losses [10], and can represent up to 10% of all transfusions performed [11]. 

The implementation of new methods, supported by innovations in medical technology which allow for the continuous measurement of hemoglobin values, would facilitate decision-making regarding transfusions, allowing them where strictly necessary and at the right moment, therefore reducing unnecessary transfusions.

The main objective of this study is to evaluate the use of the new technology to continuously measure hemoglobin in patients undergoing surgeries which involve significant blood loss. As a secondary objective, the impact in terms of the number of blood transfusions with the new technology compared to common practices will be explored and translated into costs. The cost-effectiveness of including this technology will be analyzed. The economic results will be extrapolated to the whole of the Spanish National Healthcare System (SNS) for similar surgeries. The decision of whether to implement this new technology will also be aided by the creation of a cost–benefit matrix which includes the ratio of the transfused units per patient and the price of purchasing the measurement sensors.

## 2. Materials and Methods

### 2.1. Continuous Hemoglobin Measurement Equipment

**Figure 1:**
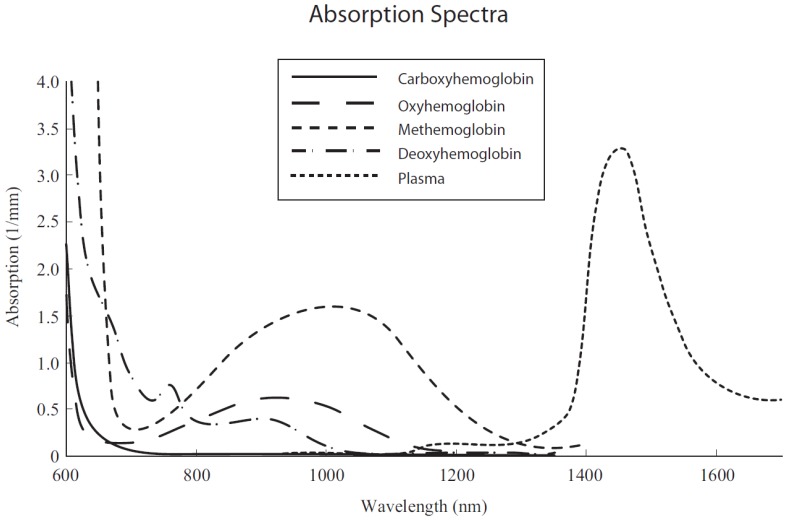
Absorption spectra of blood components [12].

Masimo^®^ (Masimo Corporation, Irvine, CA, USA) continuous hemoglobin measurement equipment Radical-7 was used with its SpHb^®^ (Masimo Corpratio registered trademark) sensor. Based on the principles of spectrophotometry and photoplethysmography, the equipment uses a multi-wavelength sensor to distinguish between oxyhemoglobin, deoxyhemoglobin, carboxyhemoglobin, methemoglobin, and plasma. The different wavelengths are expressed in nanometers in Figure 1.

### 2.2. Study Design 

An ambispective (comparative study that includes a prospective cohort and a historical control group) analytical experimental study was performed. A group of historical patients who underwent hip surgery (control group) and whose procedures did not use the new continuous hemoglobin measurement technology (SpHb^®^) was compared to a group of patients who underwent the same surgical procedure (experimental group) but which involved this technology.

The diagnosis-related group (DRG) based system is used throughout the world [13]. The most common classification in Europe is the All Patient DRG (AP-DRG) which includes 901 groups under version 27 [14]. DRGs classify patients with similar clinical characteristics and use of resources into homogeneous groups. In addition to offering a structure for accurately allocating healthcare costs for each patient type [15], DRG listings take national surgical activity data into account. This yearly public classification provided by the Spanish Ministry for Health is made up of activity data from all of the nation’s healthcare facilities.

The control group was formed of adult patients who underwent surgery on the coxofemoral joint (DRGs 210, 211, 230, 81,7 and 818) in a General University Hospital between January and December 2014. The experimental group was formed of the same type of patients, consecutive in time, for whom the continuous hemoglobin measurement sensor was used, from January to December 2015. Inclusion criteria were limited to surgeries on the aforementioned DRGs. Patients with pre-existing hematologic diseases prior to surgery and patients under 18 were excluded from both groups. Patient monitoring began upon the patient’s arrival at the surgical unit and ended once they were discharged from hospital. 

Historic transfusion ratios of the hospital facility were quantified to calculate the sample size with a percentage of 49% of patients receiving transfusions for the surgical procedures concerned in this study. Reviewing published literature and similar studies, the study published by Awada et al. [11] in 2013 was analyzed. This study was carried out on the same type of patients and procedures (patients subject to traumatology procedures), the results of which reflected a reduction in the percentage of patients having to have a transfusion by 10%. Masimo^®^ (Masimo Corporation, Irvine, California, USA), the manufacturer and distributor of the SpHb^®^ sensor, calculates saved transfusions for this type of patient to be 15%. The conservative estimated saving percentage of 12.5% was used to calculate the sample size. 

The study was approved by the hospital’s ethics committee and carried out within the framework of usual clinical practice, and therefore did not involve any of the care teams or interfere with the patient’s standard surgical procedure. Obtaining, processing, and analyzing data was performed in accordance with Spanish Organic Law 15/1999 on Personal Data Protection [16].

### 2.3. Savings on Units Transfused per Patient

To calculate the difference in the units transfused per patient, data on units used in the control group was compared to the units used in the experimental group.

### 2.4. Economic Impact

The economic impact was considered by subtracting measurement costs from the gross savings due to reducing the number of transfusions. 

Knowing the current cost of a transfusion in Spain is necessary to calculate the savings from transfusions. According to Darbá, a transfusion in Spain costs €350. It should be remembered that this cost relates to 2007, when the article was published. Without more recent studies to refer to, an approximation can be made, estimating the current cost (for 2015) as the cost from 2007 plus the corresponding accumulated increase in the Spanish Consumer Price Index (CPI) for the health sector in accordance with the ECOICOP classification as recommended by Scharff [17]. The increase in CPI for the health sector for the period 2007–2015 was 5.1%. The updated cost of a transfusion in 2015 is therefore:

€350 (2007) × 5.1% = €367.85 (2015).

This cost refers only to the transfusion cost, regarding direct costs of the red blood cell package, and indirect costs regarding storage, energy, and workforce costs. However, blood transfusions have shown adverse events (AEs) that augment morbidity and mortality [18]. Therefore, to calculate the real cost of transfusion we need to sum with Darbá’s cost those costs associated with AEs due to transfusions. The updated total cost of the transfusion unit is €371.05 [19].

The associated costs for the prospective cohort include measurement costs. These costs relate to using an individual disposable sensor for each patient to continuously measure hemoglobin (SpHb^®^). The cost of this sensor supplied by the health facility’s purchasing department was €40 for each patient. There were no additional costs relating to the measurement equipment and its maintenance, as these were included in the price of the measurement sensor.

### 2.5. Economic Impact for the Spanish National Healthcare System (SNS)

The impact on the SNS was considered as the difference between the gross savings in transfusion costs and the cost of measurement. The estimated gross economic saving for the SNS was calculated by multiplying the units saved per patient in the experimental study by the number of annual surgeries performed nationally over the period of this study. The number of annual surgeries was obtained from the DRG lists. The cost for the SNS relates to measurement costs due to using the sensor, and was calculated by multiplying the same number of patients by the price of the sensor.

### 2.6. Cost-Effectiveness Matrix

Medical information systems can extract increasingly more information from the care activity of healthcare facilities, implementing quality, effectiveness, and activity indicators. Each type of surgery performed in Spanish healthcare facilities has a different average of the number of units transfused per patient due to various factors such as the anatomical region operated on, the surgical technique, drugs used, and procedure length. Therefore, superficial, minimally invasive, and short surgeries will have almost zero units transfused per patient whereas surgeries like those analyzed for this hip surgery study, or which are invasive or lengthy, will have higher ratios of units transfused per patient. The first data entered into the matrix were the ratio of units transfused per patient depending on the type of surgeries analyzed.

Dealings between each healthcare facility and the company supplying the continuous hemoglobin measurement equipment and expendable material are private, and therefore the second lot of data entered was the various potential prices for purchasing the SpHb^®^ continuous hemoglobin measurement sensor.

To create the cost–benefit matrix for the implementation of the SpHb^®^ measurement technology, the savings obtained from reducing the number of units transfused per patient must be taken into account. These savings were classified by different types of surgeries identified by the transfusion ratio and subsequently cross-referenced with the different costs of purchasing the SpHb^®^ measurement sensor.

### 2.7. Measurement Quality

As with any new technology responsible for providing a measured value, it is necessary to know the quality of this measurement, made by the new system, compared to the standard and proven method used so far. During the surgical procedures of the experimental group, pairs of measurements were compared between the values offered by the continuous hemoglobin measurement sensor and the hemoglobin values analyzed in the laboratory by the traditional hematological analyzer.

### 2.8. Statistical Considerations

Sample size: accepting an alpha risk of 0.1 and a beta risk of 0.2 in a bilateral contrast, 108 subjects were needed to detect a difference equal to or greater than 0.125 units. It was assumed that the proportion in the reference group was 0.49. A loss to follow-up rate was estimated at 10%. The formula based on normal approximation to binominal distribution was used.

The evaluation of the impact due to the use of the new SpHb^®^ continuous hemoglobin measurement technology by Masimo^®^ (Masimo Corporation, Irvine, CA, USA) was performed by comparing the results obtained in the patients in the control group against those in the intervention group after 12 months of monitoring.

The results of the socio-demographic variables (age) and clinical variables (previous hemoglobin value) are presented as their average, standard deviation, and normal distribution. Fisher’s F-test and the *p*-value for statistical probability were used. For the comparison of homogeneous samples, those with *p* > 0.05 as a result were considered as statistically significant.

The data regarding reductions in transfusions were analyzed as the percentage of transfused patients and units transfused per patient. The results of the reduction in transfusions were translated into economic costs using the updated unitary cost of a transfusion.

Karl Pearson correlation coefficient was used to determine the quality of measurement between the SpHb^®^ sensor and the hematological analyzer. 

## 3. Results

### 3.1. Study Population

Control group: From January to December 2014 in the HUMS, 122 patients underwent hip trauma surgery. Having analyzed the clinical histories of all the patients, seven patients were excluded from the control group due to pre-existing hematologic diseases prior to surgery. Experimental group: From January to December 2015 in the HUMS, 127 patients underwent hip trauma surgery. Five patients were excluded due to pre-existing hematologic diseases prior to surgery. No patients declined to participate in the study.

Both groups were treated by the same traumatology service, performing the same surgical techniques and also by the same anesthesiology service, using the same drugs. Therefore, the transfusion criteria were exactly the same for both groups. To determine the suitability of comparing both groups, a *p*-value analysis was performed on the main socio-demographic variable (age) and the clinical variable (pre-surgery hemoglobin value). Since hemoglobin (Hb) and age distributions of both groups were normal, to determine if the variances were statistically equal we used the Fisher F-test with a result of 0.403 for the age and 0.174 for the prior Hb values (both greater than 0.05 alpha). Therefore, we could assume that groups’ variances were statistically equal. Since samples were normally distributed and population variances were statistically equal, a *p*-value could be calculated. If a usual contrast power of 0.05 (alpha) is established, it is observed that no significant differences were obtained between the control group and the experimental group given the high *p*-value result. Results are shown in Table 1. 

### 3.2. Transfusion Reduction Results

The percentage of patients undergoing a transfusion decreased from 48.7% to 45.1%, or in other words, a 7.4% reduction. The number of units transfused per patient reduced from 1.322 to 1.156, or a 12.56% decrease, which equates to a savings of 20 transfusions.

Because it is common for the same patient to receive more than one blood transfusion during surgery or in the following 24 h, a more telling sign of the effectiveness of the new technology is reflected in this latter ratio. Transfusion results of both groups are shown in Table 2.

If for the experimental group the number of units transfused per patient reflected in the control group (1.322) had stayed constant, 161.2 (122 × 1.322) transfusions would have been effected instead of the actual 141. A savings of 20 transfusions was therefore estimated by rounding down.

### 3.3. Economic Results

The total economic savings was €2541, or rather, €20.83 per patient, calculated as follows:

Estimated economic savings due to fewer transfusions:

€371.05 × 20 fewer transfusions = €7421.

Expenses incurred from measurement costs:

€40 (cost of measurement sensor) × 122 patients = €4880.

The total impact for the center is the difference between the economic savings from performing fewer transfusions and the expenses incurred from measurement: €2541 (€20.83/patient).

### 3.4. Estimated Economic Impact for the SNS

At the national level, for the same type of patients, estimated savings are €1.756 million and 13,500 fewer transfused units. 

The Spanish Ministry for Health publishes data on the lists of DRGs every year. In 2015, Spain had 81,329 cases. The number of cases was obtained by adding together the DRGs related to the type of surgical procedure as shown in Table 3.

Total cases were considered, not the adjusted cases (disregarding extreme values), given that the exclusion of cases with extreme values in DRGs for calculating indicators is inadequate [20]. 

If for the national cases reflected in Table 3 the number of units transfused per patient reflected in the control group (1.322) had remained constant, there would have been 107,516.9 transfusions. Applying the new ratio of transfused units (1.156) from using the SpHb^®^ technology, there would have been 94,016.3 transfusions. The savings is therefore approximately 13,500 transfusions. The methodology for calculating this is identical to that above:

Estimated economic savings due to fewer transfusions:

€371.05 × 13,500 fewer transfusions = €5.009 million.

Expenses incurred from measurement costs:

€40 × 81,329 patients = €3.253 million.

The total impact for the center is the difference between the economic savings due to fewer transfusions and the expenses incurred in measurement: €1.756 million. This estimated savings was calculated with the unitary cost per sensor established as the €40 provided by Masimo^®^ (Masimo Corporation, Irvine, CA, USA). 

### 3.5. Cost-Effectiveness Matrix

**Figure 2:**
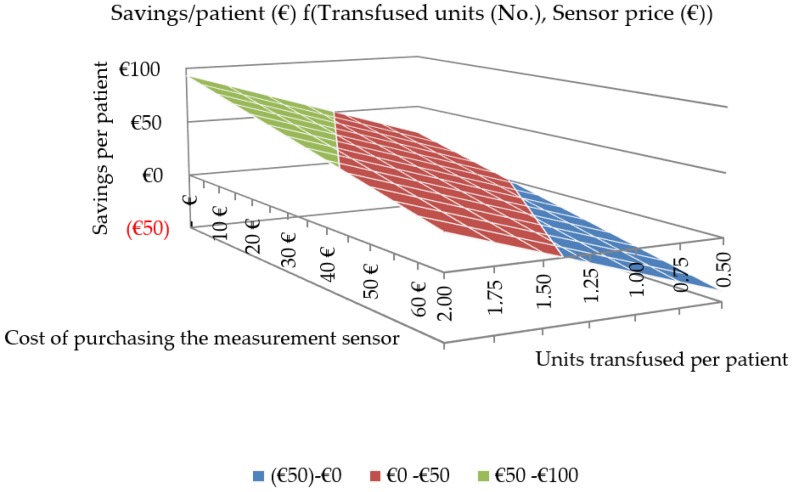
Cost-effectiveness matrix.

Table 4 and Figure 2 show the economic savings estimated per patient depending on two variables: the ratio in units transfused per patient that different types of surgeries may entail (columns) and the cost of purchasing the sensor (rows). 

### 3.6. Quality of Measurement

Overall, 209 measurement pairs were analyzed. Karl Pearson correlation coefficients were greater than 0.72 with an average difference lower than 0.78 g/dL.

## 4. Discussion

The variables analyzed in the control and experimental groups (age and prior Hb value), as well as sample size, were shown to be statistically relevant in size and homogeneity.

Savings were demonstrated in the percentage of patients requiring a transfusion as well as in the units transfused per patient thanks to the use of the SpHb^®^ measurement sensor. This savings had already been studied in surgeries with similar blood losses [11]. The data analyzed in this study were similar to those in the cited article.

For the first time in studies analyzing this new technology, the economic impact of care cost savings were added, both for the center where this study was carried out and for the entire SNS. 

Also presented for the first time is a cost-effectiveness matrix which acts as a guide for care managers when making the decision of to whether to implement this technology, based on cost-effectiveness criteria and applying the ratios and purchasing prices for each hospital facility belonging to the SNS. Given its huge purchasing volumes, the SNS can aggressively negotiate with medical technology manufacturers, obtaining more competitive purchase prices than those quoted in this article, which allows for greater economic savings than those reflected herein.

The continuous hemoglobin measurement monitor used also provides the immediate hemoglobin value of the patient and real-time trends of this value, indicating whether they are stable when they appear to be dropping, or indicating sharp increases when the perception could be of slower increases or stability. These trends allow for better clinical control of patients, and the possibility of foreseeing complications and reducing errors that are hard to detect.

The data and costs reflected are the most current possible. DRG data relates to 2015 due to the Ministry for Health publishing national care data with an almost two-year delay. For this reason, all of the costs and dates refer to this year and not the current one (2018). As the Ministry for Health provides activity data for subsequent years, the impact for the SNS can be adjusted.

It should be highlighted that when updating the cost of a transfused unit, not of all of the costs were taken into consideration. In addition to the costs related to the actual act of transfusion, blood transfusions increase the morbidity and mortality of recipients [21,22,23], therefore further increasing health and social costs. The adverse effects of transfusions can include fever, allergic reactions, viral and bacterial infections, pulmonary damage and edemas, or cardiac damage, resulting in hospital care for patients or prolonging the average length of their stay [24]. Additionally, although in a much smaller proportion, blood transfusions can cause the death of the recipient [25], bringing with it a significant social cost. The valuation of the social cost is extremely important, and various studies recommend it be included in economic evaluations [26]. Not taking the costs of morbidity and mortality associated with blood transfusions into consideration may mean that the current cost of transfusions has been undervalued, and therefore, savings may also be underestimated.

SpHb^®^ sensor technology uses seven wavelengths of light to continuously and noninvasively measure carboxyhemoglobin (SpCO^®^), methemoglobin (SpMet^®^), total hemoglobin (SpHb^®^) (SpCO^®^ and SpMet^®^ are Masimo Corporation registered trademark), pulse and oximetry, as well as providing a more reliable probe-off detection. However, the SpHb^®^ Sensor does not measure any other blood values; therefore, its utility is bound to help transfusion decisions, since the drawbacks of traditional methods mean that some transfusions are unnecessary.

The continuous hemoglobin measurement procedure based on spectrophotometry and photoplethysmography is unique and used under patent registration by the firm Masimo^®^ (Masimo Corporation, Irvine, California, USA). There are other photonic-based sensors that offer other clinical values (pulse in beats per minute and the amount of oxygen in blood). The SpHb^®^ sensor also provides these two clinical values. On the other hand, there are other measurement procedures and systems to obtain the hemoglobin value, yet all of them provide the hemoglobin value invasively and, therefore, intermittently. For example, the HemoCue^®^ is a portable system that can be used at a point of care. However, it still needs blood samples to obtain the hemoglobin value.

Biosensors based on spectrophotometry, such as the SpHb^®^ sensor analyzed in this article, augment the quality of care of patients. A recent study [27], focused on the measurement of glycemic markers for diabetes monitoring based on photonic methods, critically reviews the photonic tools that are well-suited to reagent-free marker quantitation. In the near future, research and development in photonics, spectrophotometry, and blood components’ response to different wavelengths may offer clinical values that result in better treatments while reducing healthcare costs.

## 5. Conclusions

Constant monitoring of the value of hemoglobin during surgeries with significant blood loss significantly reduces blood transfusions. Based on the reduction of transfusions by using this measurement technology, health facilities can significantly reduce their costs while improving quality of care.

## Figures and Tables

**Table 1:** *p*-Value analysis for age and prior hemoglobin (Hb) value between control and experimental groups.

Group Features	Total Patients	Average Age (years)	Standard Deviation (years)	Prior Hb Value (g/dL)	Standard Deviation (g/dL)
Control group	115	71.86	15.297	13.32	2.073
*Women*	*77*	*75.13*	*13.721*	*13.22*	*2.169*
*Men*	*38*	*65.24*	*16.338*	*13.54*	*1.871*
Experimental group	122	71.56	71.56	13.53	1.901
*Women*	*69*	*75.43*	*75.43*	*13.34*	*1.894*
*Men*	*53*	*66.51*	*66.51*	*13.78*	*1.9*
*p*-Value		0.88		0.4238	

**Table 2:** Transfusion results.

Group Features	Total Patients (No.)	Transfusions (No.)	Transfusions (%)	Units of Blood (No.)	Units per Patient (No.)
Control Group	115	56	48.7	152	1.322
*Women*	*77*	*37*	*48.05*	*124*	*1.61*
*Men*	*38*	*19*	*50*	*28*	*0.74*
Experimental Group	122	55	45.1	141	1.156
*Women*	*69*	*30*	*43.5*	*103*	*1.49*
*Men*	*53*	*25*	*47.2*	*38*	*0.72*

**Table 3:** Hip surgery cases in Spain. 2015. Source: Spanish Ministry for Health. DRG: diagnosis-related group.

DRG Code	DRG Description	Cases (No.)
210	Hip & femur procedures except major joint age >17 with complications	13,278
211	Hip & femur procedures except major joint age >17 without complications	21,765
212	Hip & femur procedures except major joint age 0–17	1608
230	Local excision & removal of int fix devices of hip and femur	3047
817	Hip replacement or review due to complications	4953
818	Hip replacement except for complications	36,678
	Total	81,329

**Table 4:** Estimated savings per patient (€) depending on units transfused (No.) and cost of purchasing the SpHb^®^ sensor (€).

		Units Transfused per Patient (No.)						
		2.00	1.75	1.50	1.25	1.00	0.75	0.50
**Cost of Purchasing the SpHb^®^ Sensor (€)**	**0**	93.2	81.6	69.9	58.3	46.6	35.0	23.3
	**5**	88.2	76.6	64.9	53.3	41.6	30.0	18.3
	**10**	83.2	71.6	59.9	48.3	36.6	25.0	13.3
	**15**	78.2	66.6	54.9	43.3	31.6	20.0	8.3
	**20**	73.2	61.6	49.9	38.3	26.6	15.0	3.3
	**25**	68.2	56.6	44.9	33.3	21.6	10.0	−1.7
	**30**	63.2	51.6	39.9	28.3	16.6	5.0	−6.7
	**35**	58.2	46.6	34.9	23.3	11.6	0.0	−11.7
	**40**	53.2	41.6	29.9	18.3	6.6	−5.0	−16.7
	**45**	48.2	36.6	24.9	13.3	1.6	−10.0	−21.7
	**50**	43.2	31.6	19.9	8.3	−3.4	−15.0	−26.7
	**55**	38.2	26.6	14.9	3.3	−8.4	−20.0	−31.7
	**60**	33.2	21.6	9.9	−1.7	−13.4	−25.0	−36.7
	**65**	28.2	16.6	4.9	−6.7	−18.4	−30.0	−41.7
